# Granulomatous filler reaction treated with adalimumab: a case report and literature review^؆^

**DOI:** 10.1016/j.abd.2025.501214

**Published:** 2025-11-01

**Authors:** Pablo Gamez-Siller, Hector Moreno-Davila, Max Oscherwitz, Rodolfo Franco-Marquez, Dionicio Angel Galarza-Delgado, Jesus Alberto Cardenas-de la Garza

**Affiliations:** a Rheumatology Department, University Hospital “Dr José Eleuterio González”, Autonomous University of Nuevo León, Monterrey, Mexico; b Master of Business Administration Program, Heersink School of Medicine, University of Alabama at Birmingham, Birmingham, Alabama, United States of America; c Pathology Department, University Hospital “Dr. José Eleuterio Gonzalez”, Autonomous University of Nuevo León, Monterrey, Mexico

Dear Editor,

Dermal fillers (DF) are one of the most common procedures performed by dermatologists, and their use continues to grow yearly.^1^ The materials used as DF include hyaluronic acid, calcium hydroxylapatite, poly-L-lactic acid, silicone, and polymethylmethacrylate (PMMA).^2^

There is no consensus on the definition of early and late adverse reactions concerning time. Mild adverse events associated with DF, as classified by the World Health Organization (WHO), include swelling, bruising, and erythema at the injection site.^3^ These effects are generally self-limited and do not require significant medical intervention. In contrast, moderate adverse events such as mycobacterial infections and granulomatous inflammatory reactions often require medical treatment.^3^

Some fillers have specific approaches for granuloma treatment, although evidence of their effectiveness varies depending on the type of filler used.^4^ Surgical extraction is rarely recommended, not only due to the possibility of filler migration but also because of significant anatomical limitations. Fillers are often present in the musculoaponeurotic system or near critical structures such as vessels, nerves, or glands, which may render excision unfeasible.^5^ In severe or recalcitrant cases, Tumor Necrosis Factor-α (TNF-α) inhibitors such as etanercept and adalimumab ‒ typically reserved for serious, treatment-resistant complications ‒ have been employed with success.^6^, ^7^, ^8^, ^9^

We present the case of a woman with a severe delayed granulomatous filler reaction with response to adalimumab and perform a review of TNF-α inhibitors used in these complications.

A 58-year-old woman presented to the dermatology clinic with an eight-year history of recurrent facial swelling. The patient's symptoms started after receiving cosmetic filler injections in the face with an unknown material by a general practitioner. At the initial examination, the patient presented with painful facial edema, erythema, and the presence of inflammatory subcutaneous nodules in the cheeks and lips (Fig. 1). The patient had received treatment with methotrexate and hydroxychloroquine for 2-years, colchicine for 1-year, and antibiotics, including doxycycline and minocycline, for 6-months without improvement. She had been employing intramuscular dexamethasone every two weeks for more than five years with irregular control. Skin biopsies showed a chronic inflammatory infiltrate composed of histiocytes and lymphocytes. Abundant translucent micro-vacuoles of silicone-like material were found inside the histocytes. Histopathology was consistent with a foreign body granuloma (Fig. 2). Ziehl-Neelsen, Gram, and PAS staining were negative. The histocytes were CD68 positive and S-100 negative. Skin biopsy cultures for bacteria, mycobacteria, and fungi were performed twice. All cultures were negative. PCR on skin biopsies was performed for the detection of *Mycobacterium tuberculosis* and atypical mycobacteria, which were also negative. A computed tomography was performed, which revealed right-sided facial edema, inflammation, and the presence of soft-tissue foreign bodies embedded in the subcutaneous tissue. No abnormalities in bones, the oral cavity, pharyngeal mucosal, or facial muscles were identified. Subcutaneous adalimumab 40 mg every two weeks was prescribed following an 80 mg loading dose. After six weeks, the patient referred complete improvement of her symptoms with full regression of subcutaneous nodules and inflammation. At the twelve-month follow-up, she continues to receive adalimumab every two weeks with complete disease control. At the 18-month follow-up, an ultrasound was performed to evaluate the DF. The ultrasound revealed in the lips and subcutaneous tissue of the malar and periorbital regions the “snowstorm” sign, which is compatible with silicone filler (Fig. 3).

**Fig. 1:**
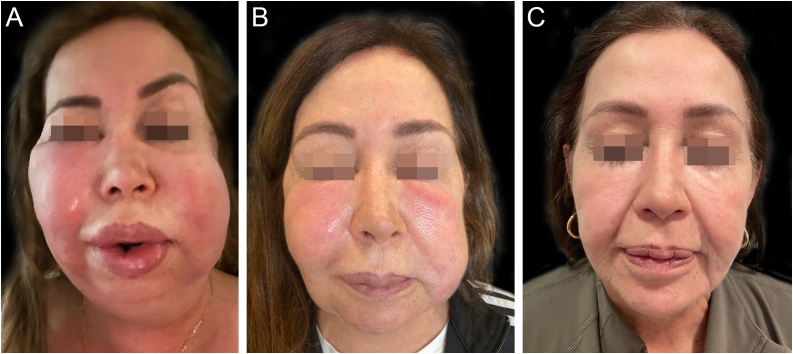
Adverse reaction to dermal filler. (A and B) Facial edema, erythema, and subcutaneous nodules in the cheeks, lips, and eyelids. (C) Patient at the one-year follow-up after starting treatment with adalimumab.

**Fig. 2:**
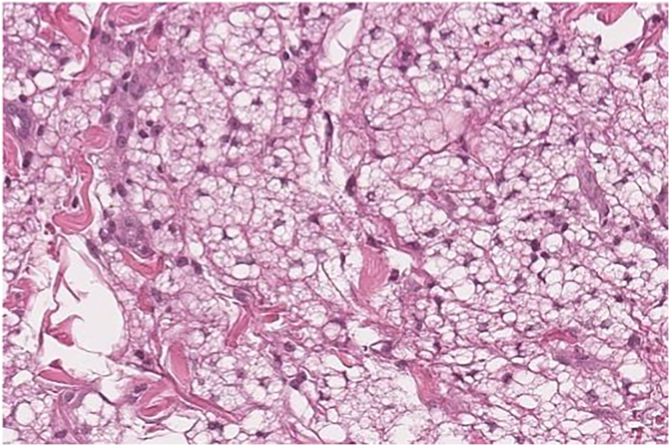
Granulomatous inflammation with lymphocytes and foamy histiocytes with abundant vacuoles (Histopathology with Hematoxylin & eosin; ×40).

**Fig. 3:**
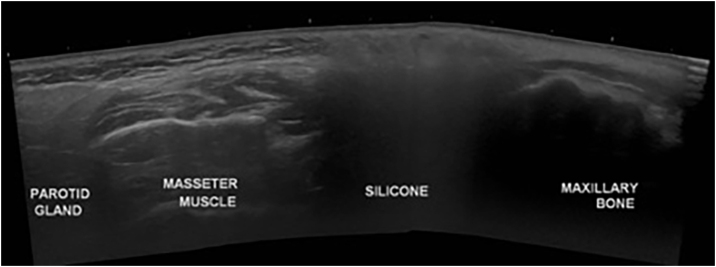
Transverse panoramic ultrasound image of the right cheek with the characteristic “snowstorm” sign between the masseter muscle and maxillary bone.

The long-term safety of adalimumab has been well established in other conditions. Annual monitoring with tuberculosis, hepatitis B, and C testing is necessary. We plan to continue treatment for at least two years and, depending on the clinical response, modify the dosage every 3-weeks.

We performed a literature review on the use of TNF-α inhibitors and adverse reactions to DF on December 20, 2024, through PubMed with the following search strategy: (adalimumab OR etanercept OR "TNF alpha inhibitors" OR "TNF inhibitors") AND (silicone OR "hyaluronic acid" OR granuloma OR dermal filler OR cosmetic).

Out of the 336 retrieved records, we identified six cases that presented an adverse reaction due to DF and were treated with a TNF-alpha inhibitor (Table 1). One of the cases full texts could not be retrieved from electronic records, and five cases were included in our analysis. The ages of the patients ranged from 36 to 78 years-old.^6^, ^7^, ^8^, ^9^ In regards to DF composition, all cases reported the use of silicone fillers.^6^, ^7^, ^8^, ^9^ Three patients had injections in the calves and buttocks area, and two patients had injections in the facial area.^6^, ^7^, ^8^, ^9^ Three patients were treated with adalimumab 40 mg subcutaneously every two weeks, and two patients with etanercept 25 mg subcutaneously twice a week.^6^, ^7^, ^8^, ^9^ In most cases, the time to improvement after starting the TNF-α inhibitor ranged from 1‒2 months with full regression at subsequent follow-up.^7^, ^8^, ^9^

**Table 1:** Articles included in the literature review of anti-TNF alpha inhibitors and adverse reactions to dermal fillers.

Author	Year	Age	Sex	Anti-TNF therapy used	Posology	Material used	Area injected	Time of response after treatment	Time to develop lesions	Adverse Reaction	Failed treatment	Follow up time and outcomes
Pasternack et al.^9^	2005	47	Woman	Etanercept	25 mg subcutaneously twice a week	Silicone	Calves and Buttocks	1 month	10 years	Erythema, swelling, induration, and tenderness of the anterior legs and ankles in the calves and buttocks	-Piperacillin and tazobactam -Trimethoprim and sulfamethoxazole. -Amikacin and imipenem -Linezolid -Minocycline	2 months and regression of lesions
Pasternack et al.^9^	2005	37	Woman	Etanercept	25 mg subcutaneously twice a week	Silicone	Buttocks	1 month	5 months	Erythema and edema of the posterior left leg and tender, subcutaneous nodules with overlying induration of the buttocks	-NSAIDs -Hydrocodone -Minocycline	2 months and complete remission
Guhan et al.^8^	2021	36	Woman	Adalimumab	40 mg every 2 weeks after an 80 mg loading dose	Silicone	Buttocks	2 months	3 years	Violaceous to pink, slightly indurated plaques on the bilateral buttocks	-Cephalexine -Betamethasone Dipropionate 0.05% -Hydroxychloroquine -Minocycline	2 months and complete remission
Silverberg et al.^6^	2022	78	Woman	Adalimumab	40 mg every 2 weeks	Silicone	Cheeks and glabella	Not mentioned	18 years	Erythematous, tender plaques on cheeks, and forehead	-Prednisone -Triamcinolone acetonide -Doxycycline -Cyclosporine -Mycophenolate mofetil	14 months and complete remission
de la Torre Gomar et al.^7^	2023	63	Woman	Adalimumab	40 mg every 2 weeks after an 80 mg loading dose	Silicone	Lips	1 month	20 years	Facial angioedema and subcutaneous erythematous nodules located on the forehead and cheeks.	-Prednisone -Allopurinol -Triamcinolone acetonide -Loratadine -Hydroxychloroquine -Dapsone	26 months and complete remission
Current Report	2024	58	Woman	Adalimumab	40 mg every 2 weeks after an 80 mg loading dose	Unknown	Cheeks and lips	2 weeks	8 years	Painful facial edema, erythema, and the presence of inflammatory subcutaneous nodules in the cheeks and lips	-Methotrexate -Colchicine -Hydroxychloroquine -Doxycycline -Minocycline	12 months and complete remission

Pasternack et al.^9^ presented two patients with erythema, edema, pain, and functional limitations after silicone injections in the calves and buttocks. Initial antibiotic treatments showed only mild improvement, but etanercept therapy produced a significant regression of symptoms within two weeks. Guhan et al.^8^ reported two women with silicone granulomas in the buttocks. The first case responded to antibiotics, while the other showed no improvement with antibiotics, hydroxychloroquine, or topical steroids. The second patient experienced complete remission after 10-weeks of adalimumab therapy. Silverberg et al.^6^ presented a case of silicone injections in the cheeks and glabella that led to bilateral indurated plaques on the buttocks. The patient initially responded to prednisone but experienced a flare during tapering. Treatments with triamcinolone, doxycycline, cyclosporine, and mycophenolate mofetil were ineffective, but administration of adalimumab was effective in the resolution and regression of the lesions after 14-months. de la Torre Gomar et al.^7^ described a patient with facial angioedema and subcutaneous erythematous nodules who showed no improvement after treatment with prednisone, allopurinol, triamcinolone, loratadine, and hydroxychloroquine. Therapy with adalimumab led to a response within a month.

The underlying immune mechanisms of granulomatous reactions to DF are not well known. The granulomatous response to biomaterials varies depending on their permanence and specific properties. Permanent, non-biodegradable materials typically induce a more persistent foreign body reaction due to their resistance to degradation, leading to sustained macrophage activity. In contrast, biodegradable materials are gradually resorbed and tend to elicit a less prolonged inflammatory response. Furthermore, the frequency and severity of granuloma formation are influenced by factors such as surface morphology and material fragmentation, with irregular or particulate biomaterials provoking stronger inflammatory reactions.^10^

The inability to phagocytose large foreign particles may result in chronic inflammation and granuloma formation. These granulomas are composed of macrophages and lymphocytes that secrete pro-inflammatory cytokines, including TNF-alpha, interferon-gamma, and interleukin 12. Additionally, fillers may act as adjuvants, amplifying the innate immune response and triggering the release of Th1 inflammatory cytokines. Permanent fillers, such as liquid silicone and polymethylmethacrylate, are highly susceptible to chronic inflammation, infections, abscess formation, and migration, even years after injection. In contrast, non-permanent fillers like hyaluronic acid are generally safer but can still cause granulomatous reactions. It is crucial to rule out underlying infections by bacteria, mycobacteria, or fungi in all cases of chronic reaction to dermal fillers.^11^ When the substance is unknown, it is essential to determine whether it was compounded and identify the professional responsible for the procedure.

TNF-alpha inhibitors act by blocking circulating cell-bound TNF. Through this mechanism, it has an inhibitory action on macrophages and may aid in controlling granuloma formation.^11^ After this patient failed to respond to multiple treatments (steroid, methotrexate, hydroxychloroquine, colchicine, and antibiotics), the use of a TNF-alpha inhibitor was employed as an alternative, previously reported to be effective in a few case reports.^6^, ^7^, ^8^, ^9^ Rarely, these drugs may present paradoxical granuloma formation, and this should be considered by clinicians. Granulomatous reactions due to non-permanent fillers such as hyaluronic acid may resolve spontaneously or be treated with other treatment options, including hyaluronidase or, in select cases, surgery. The effect of TNF-alpha inhibitors on non-permanent fillers is not well known, and further research is needed to determine the best treatment strategy.

As a limitation, our literature review comprises five case reports, which are insufficient to draw definitive conclusions regarding the overall efficacy of TNF-alpha inhibitors in managing granulomatous reactions to DF. Further research is necessary to determine the long-term safety and efficacy of TNF-alpha inhibitors in such cases.

In conclusion, we report a patient with a one-year follow-up and remission of granulomatous inflammatory reaction due to DF successfully treated with adalimumab. Managing these lesions can be challenging, and physicians should consider TNF-α inhibitors as a possible treatment for recalcitrant cases.

## ORCID ID

Hector Moreno-Davila: 0009-0005-4551-6224

Max Oscherwitz: 0000-0003-4572-5032

Dionicio Angel Galarza-Delgado: 0000-0001-9714-2109

Jesus Alberto Cardenas-de la Garza: 0000-0002-5099-0079

## Financial support

This research did not receive any specific grant from funding agencies in the public, commercial, or not-for-profit sectors.

## Authors' contributions

Pablo Gámez-Siller: Data collection, literature review, and drafting of the manuscript.

Héctor Moreno-Dávila: Conceptualization and critical revision of the manuscript.

Max Oscherwitz: Statistical analysis and interpretation of data.

Rodolfo Franco-Márquez: Histopathological analysis and figure preparation.

Dionicio Ángel Galarza-Delgado: Supervision and final approval of the manuscript.

Jesús Alberto Cárdenas-de la Garza: Study design, corresponding author, and manuscript revision.

## Research data availability

Does not apply.

## Conflicts of interest

None declared.
